# Telomere Attrition and Clonal Hematopoiesis of Indeterminate Potential in Cardiovascular Disease

**DOI:** 10.3390/ijms22189867

**Published:** 2021-09-13

**Authors:** Yi-Chun Huang, Chao-Yung Wang

**Affiliations:** 1 Division of Cardiology, Chang Gung Memorial Hospital, Linkou Medical Center, Taoyuan City 33305, Taiwan; twkodesamurai@gmail.com; 2 School of Medicine, College of Medicine, Chang Gung University, Taoyuan City 33302, Taiwan; 3 Institute of Cellular and System Medicine, National Health Research Institutes, Zhunan 35053, Taiwan; 4 Department of Medical Science, National Tsing Hua University, Hsinchu 30013, Taiwan

**Keywords:** atherosclerosis, clonal hematopoiesis of indeterminate potential, telomere, TCA axis

## Abstract

Clinical evidence suggests that conventional cardiovascular disease (CVD) risk factors cannot explain all CVD incidences. Recent studies have shown that telomere attrition, clonal hematopoiesis of indeterminate potential (CHIP), and atherosclerosis (telomere–CHIP–atherosclerosis, TCA) evolve to play a crucial role in CVD. Telomere dynamics and telomerase have an important relationship with age-related CVD. Telomere attrition is associated with CHIP. CHIP is commonly observed in elderly patients. It is characterized by an increase in blood cell clones with somatic mutations, resulting in an increased risk of hematological cancer and atherosclerotic CVD. The most common gene mutations are *DNA methyltransferase 3 alpha (DNMT3A)*, *Tet methylcytosine dioxygenase 2 (TET2)*, and *additional sex combs-like 1 (ASXL1)*. Telomeres, CHIP, and atherosclerosis increase chronic inflammation and proinflammatory cytokine expression. Currently, their epidemiology and detailed mechanisms related to the TCA axis remain incompletely understood. In this article, we reviewed recent research results regarding the development of telomeres and CHIP and their relationship with atherosclerotic CVD.

## 1. Introduction

With age, genomic instability and acquired somatic mutations of hematopoietic stem cells (HSCs) gradually occur, and some clones disproportionately occupy the bone marrow and peripheral blood cells. The clonal expansion of mutant cells is associated with increased risk of hematologic disease [1]. People with increased blood cell clones without hematological malignancy are considered to have clonal hematopoiesis of indeterminate potential (CHIP). CHIP is defined as somatic mutations of a leukemia-associated gene with variant allele frequency (VAF) ≥ 2%, normal peripheral blood counts, and no clinical or pathological evidence of World Health Organization (WHO)-defined hematologic malignancy neoplasm [2]. CHIP was observed in >10% of people aged >70 years [3]. Age-related CHIP is associated with an increase in hematologic cancer risk, and people with CHIP have high all-cause mortality. The increased mortality risk is mostly due to increased cardiovascular diseases (CVDs) [3]. The exact mechanism by which CHIP leads to high CVD occurrence is under investigation. According to current research, chronic inflammation through several specific pathways may be crucial mechanisms of CHIP-related atherosclerosis [4]. Targeting these specific inflammatory pathways may be the focus of future research.

Telomeres are double-stranded repeats of G-rich tandem DNA sequences that gradually shorten with each cell division. The main function of telomeres is to protect crucial genetic information in the cell from being lost during cell division. Each time the cell divides, the ends of the chromosomes shorten by 25–200 bases [5,6,7]. Because telomeres protect the terminal regions of chromosomal DNA, the telomeres ensure the retention of crucial genetic information even after cell division. As the cell undergoes multiple divisions, the telomere length decreases. When the telomere reaches a critical length, the cell can no longer divide, and undergoes the physiological mechanism of cellular senescence. Telomerase can synthesize DNA end sequences and extend telomeres, playing an important role in maintaining telomere dynamics and preventing DNA damage. Recent studies have shown that inflammation, oxidative stress, and age accelerate telomere shortening [8,9,10]. Telomere shortening and telomerase dysfunction both play crucial roles in the pathophysiological mechanism of aging-associated CVD [11,12,13]. Telomere therapy for CVD became popular because of population aging and growing evidence of the role of telomere dysfunction in cardiovascular physiology [14].

Cardiovascular treatments focus on traditional cardiovascular risk factors, which include hypertension, diabetes, smoking, dyslipidemia, and obesity. However, many patients develop CVDs despite the absence of these traditional risk factors [15,16,17,18,19]. Thus, our understanding of CVD pathophysiology remains incomplete. Clinical observations have revealed significant associations between decrease in telomere length and increase in CHIP. Both these phenomena are related to increased CVD risk. CHIP is an independent risk factor for CVD. Furthermore, studies have suggested that the manipulation of telomeres and telomerase is a treatment strategy for CVD. Moreover, telomere dynamics may play a role in CHIP development [20,21,22]. This review focuses on the current evidence supporting the relationships between telomeres, CHIP, and CVD.

## 2. Telomere and Telomerase in CVD

A telomere is a repetitive sequence (TTAGGG)n located at the end of the chromosomal DNA of eukaryotic cells. The functions of telomeres and telomerase involve ensuring the complete replication of chromosomal DNA and prevention of chromosomal end damage. Telomerase consists of a telomerase RNA component (TERC) and a catalytic telomerase enzyme reverse transcriptase (TERT) [5]. Telomerase stabilizes telomere length. Telomerase activity is precisely controlled through multiple mechanisms, which ensure adequate telomere length homeostasis [23]. Although the telomere length varies by individual, the length of the human telomere is approximately 10–15 kb. At each cell division, the telomere DNA shortens by 50–100 base pairs due to incomplete replication at the 3′ end. This mechanism leads to telomere shortening [6]. When the telomere is shortened to a critical point, genomic instability occurs and the DNA repair system is activated to cause replicative arrest, senescence, and cell death [8,24,25]. By contrast, increased telomere length is associated with the risk of various cancers [26,27]. Telomere length varies slightly among different tissues [28,29]. Peripheral leukocyte telomere length (LTL) can be easily determined in humans and is significantly associated with immunologic system aging and CVD risk, irrespective of traditional risk factors [30,31]. LTL gradually shortens with age and is considered the biological molecular clock [32]. At present, many detection methods are available for detecting telomere length. Among them, quantitative polymerase chain reaction (PCR) is a high-throughput and low-cost method. It only generates the mean telomere length without providing the telomere length distribution in the DNA sample. The single telomere length analysis and telomere shortest length assay are relatively new methods of measuring single-telomere data based on a combination of PCR and Southern blot [33,34].

**Figure 1:**
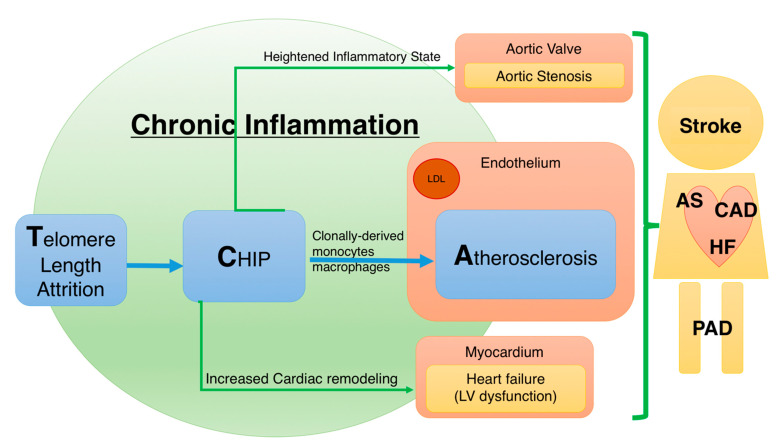
Telomere–CHIP–atherosclerosis (TCA) axis and cardiovascular disease. The TCA axis represents a novel cardiovascular treatment target in addition to traditional risk factors. With aging and telomere length attrition, hematopoietic stem cells develop clonal hematopoiesis of indeterminate potential (CHIP). CHIP then further aggravates chronic inflammation through multiple CHIP-related mutant gene signaling pathways, including *Tet methylcytosine dioxygenase 2* (*TET2*), *additional sex combs-like 1* (*ASXL1*), *Janus kinase 2* (*JAK2*), and *DNA methyltransferase 3 alpha* (*DNMT3A*). The affected cells, including macrophages, mast cells, and T cells, further increase the risk of atherosclerosis, coronary artery disease (CAD), aortic stenosis (AS), heart failure (HF), and possibly peripheral arterial occlusive diseases (PAD).

Many large epidemiological studies have supported the relationship between telomere length and CVD [12,13,35,36]. CVD and telomere shortening have many common risk factors, including diabetes mellitus, hypertension, dyslipidemia, obesity, and smoking. Furthermore, excessive oxidative stress and chronic inflammation are important factors that contribute to telomere shortening and CVD [37,38]. In addition, disrupted circadian rhythm has been confirmed to shorten telomere length and increase CVD risk [39,40,41,42]. Moreover, diets high in omega-3 fatty acid [43] and lifestyle modification with exercise [44,45] can reduce the rates of telomere shortening and CVD. In addition to these common risk factors for telomere length shortening and CVD, current clinical evidence suggests that telomeres play a crucial role in atherosclerotic CVD (Figure 1). The West of Scotland Coronary Prevention Study (WOSCOPS) showed that the mean LTL is a predictor of future coronary heart disease. In WOSCOPS, the mean telomere length decreased with age by 9% per decade. Individuals in the lowest tertiles of LTL had almost two times the risk of coronary artery events compared with those in the highest tertiles (WOSCOPS) [12]. In 2014, a meta-analysis of 24 prospective and retrospective studies that included 43,725 participants revealed that a shorter LTL is associated with a pooled relative risk of coronary heart disease of 1.54 compared with a longer LTL [13]. Virtual histology intravascular ultrasound revealed that increased proinflammatory activity in high-risk unstable plaque frequently occurs in patients with short LTL [46]. Moreover, a small clinical study demonstrated through optical coherence tomography that a shorter LTL indicated a higher percentage of uncovered stent struts [47]. Furthermore, telomere length is shorter than usual in patients with congestive heart failure [48,49]. In a study involving 620 patients with congestive heart failure, the relative telomere length was nearly 60% that of the normal participants and was significantly correlated with their New York Heart Association function class status [50].

Studies have shown that mice with genetic *TERC* or *TERT* knockout have progressively shorter telomeres with the pathophysiological phenomena of cardiovascular dysfunction, aging, and early death. Telomere shortening in late-generation *TERC* knockout mice results in decreased cardiomyocyte proliferation, cardiac dilatation, and heart failure with p53 upregulation [48]. These cardiac changes can be delayed through telomerase re-expression [51]. Telomere shortening results in immune cell dysfunction and chronic inflammation [52,53]. In *TERC-* or *TERT*-knockout mice, proinflammatory cytokines including interleukin (IL)-6, chemokine (C-X-C motif) ligand 16, and tumor necrosis factor-α are enhanced [54]. Moreover, increased inflammation and atherosclerosis are observed in late-generation *TERT* or *TERC* knockout mice. Chronic inflammation further increases oxidative stress and induces telomere dysfunction [37,38]. Although there is already abundant evidence to support the relationship between the telomere system and chronic inflammation in the cardiovascular system. There are still several studies that argue against this possibility. Past studies have shown that telomere attrition as a mechanism for restricting atheroma progression in hypercholesterolemic mice [55]. Some people have reorganized the current evidence and various experiments to question the causal relationship between telomere attrition and CVD is not clear enough [56]. More research focusing on this direct connection is needed in the future.

Experimental therapies targeting telomeres or telomerase in mice have shown promising results in CVDs. Telomerase gene therapy was first achieved through delivering mouse *TERT* with an adeno-associated virus into young and old mice. In the experiment, elongated telomeres, extended lifespans, and delayed age-associated pathologies were observed in both the age groups. The treatment group with the *TERT* adeno-associated virus did not have higher cancer incidences than those of the placebo group [57]. Using this *TERT* adeno-associated technique in mice with myocardial infarction conferred significant cardioprotection with less cardiac dilation, improved ventricular function, and smaller infarct scars compared with the control group [58]. The modified messenger RNA encoding *TERT* delivered in human fibroblasts also temporarily increases telomerase activity and extends telomere length. Research on the clinical impacts of telomere therapy on myocardial infarction is still in progress [59]. 

Some drugs used in CVD treatment are related to the regulation of telomerase activity. Statins (3-hydroxy-3-methylglutaryl coenzyme A reductase inhibitors) can prevent atherosclerotic plaque formation through multiple mechanisms [60]. A cross-sectional analysis of data from the U.S. National Health and Nutrition and Nutrition Examination Survey 1999–2002 showed that statin use for extended periods may be associated with an increase in telomere length [61]. Furthermore, statins can increase telomerase through the adjustment of the TRF2 of endothelial cells and activity of endothelial progenitor cells (EPCs) [62]. Telomere repeat binding factor 2(TRF2) is part of the Shelterin complex used to protect telomeres and regulate telomerase activity. Both angiotensin II receptor blockers and angiotensin-converting enzyme inhibitors can protect EPCs from senescence and dysfunction through telomere crosstalk [63,64]. However, whether using these drugs in actual clinical practice can slow telomere shortening requires further research [65]. In an animal experiment, peroxisome proliferator-activated receptor agonists (PPARs, e.g., pioglitazone) increased telomerase activity and *TRF2* expression [66]. Moreover, PPARs has shown to reduce senescence markers, p16, cell-cycle checkpoint kinase 2, and p53 [67,68]. TA-65, a bioactive molecule extracted from *Astragalus membranaceus*, is regarded a low-potency telomerase activator [69]. Randomized, double-blind, and placebo-controlled clinical trials have shown that TA-65 treatment increases high-density lipoprotein cholesterol and reduces c-reactive protein in patients with metabolic syndrome and elongates telomeres [70,71]. Sex hormones are believed to activate *TERT* transcription. In the past, the mechanism by which androgen therapy can be used in aplastic anemia has been unclear, but reports have indicated that androgen-induced effects are achieved through upregulated telomerase activity [72,73].

## 3. Relationships between CHIP and CVDs

Clonal hematopoiesis is an aging phenomenon in the hematologic system. Aging is associated with increases in somatic mutations in many tissues, including the spontaneous deamination of 5-methylcytosine, insertions and deletions, replication errors with base changes, and large structural variations. Considering that the somatic mutation rate of each HSC is one exonic mutation per decade of life, a 70-year-old person would have a significant number of mutations per gene. According to natural selection, HSCs or blood progenitor cells acquiring certain somatic mutations with aging will form a genetically distinct clone of blood cells. If the clone does not overgrow to form hematologic malignancies, it will persist in aged people and become prevalent. A similar phenomenon was observed in the skin and esophagus [74,75].

Clonal hematopoiesis prevalence increases with age. In healthy individuals < 40 years old, the somatic mutation clone prevalence is <1%. In individuals aged >70 years, >10% have a detectable clone. The percentage of these mutated clones varies from 0.01% to >18% depending on the detection methods used in clinical trials [3,75]. However, for determining the clinical impact of the clonal percentage, further investigations are required. Currently, no evidence supports that a larger clonal size corresponds to higher physiological disruption or changes.

**Figure 2:**
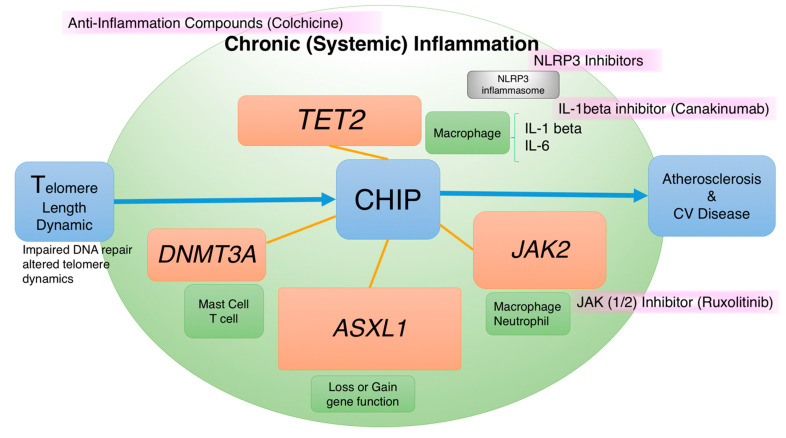
Telomere–CHIP–atherosclerosis (TCA) axis, somatic mutations, and potential therapeutic options. Four major mutant genes identified in patients with CHIP, namely *TET2*, *ASXL1*, *JAK2*, and *DNMT3A*. Here, we highlight the possible therapeutic compounds targeting each CHIP-related mutant gene pathway. The general anti-inflammatory agents are colchicine, TET2-related NOD-, LRR-, and pyrin domain-containing protein 3 (NLRP3) inhibitors targeting NLRP3 inflammasome, IL-1β inhibitor (canakinumab), and JAK2-related JAK(1/2) inhibitor (ruxolitinib).

Multiple studies have shown that CHIP is associated with a >30% increase in mortality risk. The increase in CHIP-related mortality is not due to cancer but to the increase in cardiovascular events [1,3,76]. Nested case–control analyses of two prospective cohorts revealed that CHIP carriers had nearly twice the risk of early myocardial infarction and stroke compared with noncarriers [77]. Another retrospective case–control study showed that individuals < 50 years old with CHIP have a four times higher risk of early-onset myocardial infarction [78,79,80]. Moreover, higher VAF and CHIP are associated with coronary artery calcification [77]. VAF is the percentage of sequence reads observed matching a specific DNA variant divided by the overall coverage at that locus. Clinically, because NGS provides a near random sample, VAF is thus a surrogate measure of the proportion of DNA molecules in the original specimen carrying the variant [81]. In patients with congestive heart failure, CHIP is associated with the progression and poor prognosis of congestive heart failure. For patients with ischemic heart failure and reduced left ventricular ejection fraction, the presence of CHIP is associated with adverse outcomes and increased cardiovascular events [82,83]. In some registry data, certain percentage of heart failure patients whose cause of death is related to hematologic cancer. Some studies also show that patients with advanced heart failure prone to develop cancers. This implies that we should have further research on the relationship of abnormal heart function, CHIP, and tumor growth [84,85]. Moreover, clonal hematopoiesis plays an important role in another age-associated disease, severe aortic stenosis. Patients of all age groups with severe aortic stenosis have considerably higher CHIP. Among 279 patients undergoing transcatheter aortic valve implantation (TAVI), somatic *DNA methyltransferase 3 alpha* (*DNMT3A*) or *Tet methylcytosine dioxygenase 2* (*TET2*) CHIP-driver mutations were detected in 93 out of 279 patients (33.3%). These patients with CHIP were associated with increased mortality in the first 8 months after TAVI [86]. More than 100 mutation driver genes are associated with CHIP. The most common mutations observed are *DNMT3A*, *TET2*, and *ASXL1*. Although *JAK*2 mutation is relatively less common, it has a disproportionate impact on the risk of CVD in clinical practice. (Figure 2) [76,87]. *TET2* is a multifaceted transcriptional regulator that can promote both transcriptional activation and repression according to the molecular and cytological environment. In mice, *TET2*-deficient bone marrow-transplanted cells expanded in the blood and accelerated atherosclerosis. Myeloid-specific *TET2* ablation is sufficient to promote atherosclerosis, indicating that macrophages play a role in these *TET2*-deficient cells [88]. Moreover, *TET2* deficiency in mice aggravated heart failure and exacerbated pathological cardiac remodeling [89,90]. *TET2* mutation associated clonal hematopoiesis is also reported to aggravates insulin resistance in mice, establishing a causal relationship between *TET2* mutation and type 2 diabetes mellitus [91]. Enhanced atherosclerosis with *TET2* deficiency in macrophages is mediated through increasing proinflammatory signaling, including IL-1β, IL-6, and NOD-, LRR-, and the pyrin domain-containing protein 3 (NLRP3) inflammasome complex [92]. *TET2* deficiency contributes to IL-1β production in macrophages through NLRP3 enhancement. An experiment with mice revealed that pharmacological blockade of NLRP3 inflammasome-mediated IL-1β secretion increased atheroprotective effects [88]. The association between IL-1β overproduction by *TET2* mutation and increased atherosclerosis is supported by the results of the Canakinumab Anti-Inflammatory Thrombosis Outcomes Study (CANTOS) trial. In the CANTOS trial, IL-1β blockade significantly reduced recurrent cardiovascular events in patients with previous myocardial infarction [93,94]. The role of the IL-1β monoclonal antibody in reducing cardiovascular events is independent of lipid-level reduction. A similar concept, whereby anti-inflammatory therapy can improve cardiovascular outcomes, has been supported in studies related to colchicine. In 2019, a randomized and double-blind trial, including 4745 patients with recent myocardial infarction, showed that a low dose of colchicine can effectively reduce ischemic cardiovascular events compared with a placebo [95].

*DNMT3A* deficiency of bone marrow-transplanted cells is also associated with increased cardiac fibrosis and worse cardiac function following angiotensin II infusion. *DNMT3A* augments inflammatory pathways involving macrophage accumulation in the heart and proinflammatory molecules IL-6 and CXCL5 [96]. *DNMT3A* plays a crucial role in modulating mast cell responses [97]. Furthermore, *DNMT3A* is a T-cell receptor-related regulator of Th1 and Th2 differentiation and is involved in interferon-γ expression [98]. Recent studies have also mentioned that circulating monocytes and T cells in heart failure patients may cause aggravation of the inflammatory response and worsen chronic heart failure [99]. 

*JAK2* is a tyrosine kinase which mainly plays a role in signaling transmission. It is found in patients with polycythemia vera and essential thrombocytopenia [100]. Such patients have a high risk of thrombosis and CVD. *JAK2* is involved in erythropoietin and thrombopoietin pathways [101,102,103]. Mice receiving *JAK2* mutant bone marrow transplant cells develop accelerated heart failure [104]. *JAK2* mutation showed increased expression of absent in melanoma 2 (AIM2), DNA replication stress, oxidative DNA damage, and multiple inflammatory pathway, thereby aggravating atherosclerosis [105,106,107]. Clinically, a *JAK*1/2 inhibitor, ruxolitinib, was proven to be helpful in controlling hematocrit, reducing spleen volume, and improving symptoms in patients with polycythemia vera compared with the standard treatment [108]. Adequate hematocrit control significantly reduces cardiovascular death and major thrombosis [109]. Clinical evidence suggests that *JAK*1/2 inhibitor is a potential therapeutic target, and it deserves more specific research in the future.

*ASXL1* mutation in mouse HSCs induces age-related expansion through overactive Akt/mammalian target of rapamycin (mTOR) signaling. *ASXL1* cooperates with its partner protein BAP1 to deubiquitinate and activate Akt. The mTOR inhibitor rapamycin, which has previously been shown to prolong murine life span, ameliorates dysregulated hematopoiesis in aged *ASXL1* mutant mice [110,111].

Although the association between gene mutations and CHIP has been established, a large knowledge gap exists regarding the detailed molecular biological mechanism. The exact impact of CHIP on human health is still under intense investigation. The scientific debate on directionality in the association between clonal hematopoiesis and CVD is still ongoing. Through mathematical models, some studies have pointed out that perhaps reverse causality (that is, atherosclerosis accelerates clonal hematopoiesis) could explain the clonal hematopoiesis–CVD association [112]. Several ongoing studies focused on CHIP treatment will reveal the potential clinical impact of CHIP. First, chronic IL-1 drives HSCs toward precocious myeloid differentiation, whereas *TET2* mutation may lead to IL-1 overproduction. Whether IL-1 blockade can reverse or limit clonal expansion is critical in future cardiovascular therapeutic road maps [93,94]. Research has been focused on the role of metabolism and several metabolites in CHIP development and treatment. In mouse experiments, vitamin C restored *TET2* activity in HSCs [113]. Crosstalk between the immune system and metabolism during CHIP development and atherosclerosis is also an intensely studied field. Many large-scale studies on the clinical impact of CHIP are underway to understand CHIP and related diseases and identify the specific gene mutation and associated disease development. Several prospective cohort studies on CHIP analyses are ongoing, including the Atherosclerosis Risk in Communities Study and UK Biobank [114,115,116,117]. These analyses will help us to identify the dose–response relationship between CHIP clone size and its effect on atherosclerotic CVD. The relationships between CHIP and other age-related diseases are also under investigation. For example, Alzheimer’s disease and related cognitive decline are being studied to determine whether CHIP plays a significant role in these diseases [118,119].

Despite strong evidence supporting the importance of CHIP in cardiovascular patients, no clear guideline suggests when and how to test CHIP in patients. We reasoned that several factors can be considered to study CHIP. First, in patients > 65 years old with coronary artery disease or acute coronary syndrome, no traditional risk factors or early-onset CVD were observed. Second, identified CHIP mutations will affect clinical decisions. For example, in patients with *TET2* mutation and IL-1β mutation, IL-1β blockade may serve as a viable therapeutic agent. Third, future clinical trials may confirm that CHIP clone sizes are related to cardiovascular risks. In addition to the traditional risk factors, the CHIP clone sizes and CHIP-related genes can be considered CV risk factors. These clinically important issues urgently require more research and evidence to guide decision making.

Currently, diagnosing and surveilling CHIP pose a clinical challenge for both patients and cardiologists. CHIP is detected through elective whole-genome sequencing during clinical trials, genome sequencing for studying cardiovascular traditional risks, genome sequencing in organ donors, or a hematologic or solid tumor. Strategic and systemic evaluations of cost-effective methods for CHIP detection will be required in the future.

## 4. Telomere-CHIP-Atherosclerosis Related Pathway

The Framingham Study that began in 1948 advanced our understanding on standard modifiable cardiovascular risk factors [120] (SMuRFs; hypertension, diabetes, hypercholesterolemia, and smoking). In the past few decades, research and treatment related to these traditional factors have been successful. However, even after these risk factors have been corrected and improved clinically, mortality trends remain steady in many countries. Many patients with CVDs have no identifiable traditional cardiovascular risk factors. This observation implies that blind spots remain in understanding of CVDs. Cancer and CVDs are the two main causes of mortality in the general population, and their incidences increase with age [15,16,17,18,19,121]. Their mutual association is gradually being recognized through CHIP and telomere signaling.

Genetic association studies have repeatedly shown that CHIP has polygenic and inherited risk [122]. Its associations with the *TERT* locus have been replicated in numerous clinical trials. Clonal hematopoiesis is strongly correlated with mosaic loss of the Y chromosome, which indicates a significant association between telomere biology and CHIP [76]. After high-coverage whole-genome sequences from 97,691 participants with 4229 individuals with CHIP were analyzed, a common germline variant in the *TERT* intron was identified to be significantly associated with CHIP. This result highlights the importance of telomere and telomerase function, which can shape somatic variation in HSCs. Other whole-genome sequencing and genome-wide association studies have revealed that CHIP carriers have a relatively short LTL and often carry a genetic variant of the related telomerase gene [123]. Dyskeratosis congenita is a rare inherited disease and a short telomere syndrome. Other diseases considered to be short telomere syndromes include Hoyeraal–Hreidarsson syndrome, Revesz syndrome, cerebroretinal microangiopathy with calcifications and cysts, aplastic anemia, and Fanconi anemia. Short telomere syndromes, also known as telomeropathies, are characterized by genetic mutations leading to shortened telomere length, which ultimately leads to accelerated cell turnover and disease in multiple organs. Organs that are often involved include the skin, bone marrow, lungs, and gastrointestinal tract [124,125]. Clonal hematopoiesis in people with short telomere syndromes is common. The majority of patients with dyskeratosis congenita have clonal hematopoiesis, as evidenced through skewed X-inactivation [126].

Aging, as well as exposure to various environmental factors including radiation, smoking, and air pollution, results in gradual leukocyte telomere attrition [127]. Telomere shortening and telomerase dysfunction can lead to genomic instability and ultimately to CHIP. Currently, clinical evidence supports the relationship between telomere dysfunction and CHIP. However, the direct association between telomere attrition and CHIP must be confirmed with more in-depth research. Moreover, how CHIP interacts with the complex physiology of atherosclerosis is still a subject for more comprehensive study. The telomere–CHIP–atherosclerosis (TCA) axis (Figure 1) is a new and evolving concept. There are still very limited data on the interplay between telomere biology and clonal hematopoiesis. Accumulating evidence will reveal that whether therapeutics targeting the TCA axis will further protect cardiovascular patients. Elderly patients without traditional risk factors would benefit the most from TCA-related therapy in future [21].

## 5. Conclusions

In the past 10 years, understanding and treatment of CVD have been constantly evolving. Both drugs and interventional treatments have advanced significantly. However, overall outcome improvement has stagnated. Moreover, some patients are unable to receive precise and personalized preventive treatment because they do not have SMuRFs. CHIP emergence and the discovery of its association with the TCA axis provide a new direction for cardiological research. Furthermore, this new concept solves many problems. The limited understanding of detailed mechanisms involved in CHIP makes appropriate risk stratification of patients with CHIP difficult. Furthermore, the optimal method of properly monitoring and following up with patients with CHIP is unknown. Behavioral, lifestyle, environmental, and heritable factors that affect CHIP remain unclear. More cellular or animal model studies are warranted to simulate the gradual and long-term processes of telomere attrition and increase understanding of TCA signaling.

## Data Availability

Not applicable.

## Abbreviations

*AIM2*	*absent in melanoma 2*
*ASXL1*	*additional sex combs-like-1*
CANTOS	Canakinumab Anti-Inflammatory Thrombosis Outcomes Study
CHIP	Clonal hematopoiesis of indetermenate potential
CVD	Cardiovascular disease
*DNMT3A*	*DNA methyltransferase 3 alpha*
EPCs	Endothelial progenitor cells
HSCs	hematopoietic stem cells
LTL	Leukocyte telomere length
*NLRP3*	*NOD-, LRR-, and the pyrin domain-containing protein 3*
PPARs	Peroxisome proliferator-activated receptor agnoists
SMuRFs	Standard modifiable cardiovascular risk factors
TAVI	Transcatheter aortic valve implantation
TCA	Telomere-CHIP-atherosclerosis
TERC	Telomerase consists of a telomerase RNA component
*TERT*	*Telomere length homeostasis*
*TET2*	*Tet methylcytosine dioxygenase 2*
*TRF2*	*Telomere repeat binding factor 2*
VAF	variant allele frequency
WHO	World Health Organization
WOSCOPS	The West of Scotland Coronary Prevention Study
